# Antimicrobial Polymeric Materials with Quaternary Ammonium and Phosphonium Salts

**DOI:** 10.3390/ijms16023626

**Published:** 2015-02-06

**Authors:** Yan Xue, Huining Xiao, Yi Zhang

**Affiliations:** 1 Department of Chemical Engineering, University of New Brunswick, Fredericton, NB E3B 5A3, Canada; E-Mail: xiaoxue8515@126.com; 2 School of Chemistry and Chemical Engineering, Southwest Petroleum University, Chengdu 610500, China; 3 School of Environment Science & Engineering, North China Electric Power University, Baoding 071003, China; E-Mail: zyi0251@gmail.com

**Keywords:** antimicrobial, cationic polymer, quaternary ammonium salt, quaternary phosphonium salt

## Abstract

Polymeric materials containing quaternary ammonium and/or phosphonium salts have been extensively studied and applied to a variety of antimicrobial-relevant areas. With various architectures, polymeric quaternary ammonium/phosphonium salts were prepared using different approaches, exhibiting different antimicrobial activities and potential applications. This review focuses on the state of the art of antimicrobial polymers with quaternary ammonium/phosphonium salts. In particular, it discusses the structure and synthesis method, mechanisms of antimicrobial action, and the comparison of antimicrobial performance between these two kinds of polymers.

## 1. Introduction

Microbial pathogens, which can cause infections and diseases in animals, plants, and human beings, have long been a threat to human health and social development. As one of the leading causes of death worldwide, outbreaks of infectious diseases triggered by bacteria, viruses, and fungi lead to over one-fourth of global deaths annually [1,2,3]. Since microorganisms exist everywhere and can be spread through air, water and food, *etc.* the control and prevention of microbial infections becomes a daunting challenge. To combat with microbial pathogens, all kinds of antimicrobial agents, including antibiotics, disinfectants and antiseptics, have been developed substantially. However, the widespread and injudicious use of antibiotics and disinfectants has induced the emergence of new strains of antimicrobial-resistant microorganisms, leading to dramatically increased difficulties in the antimicrobial issue [4,5,6,7]. A data analysis from U.S. Centers for Disease Control and Prevention (CDC) reported that each year in the United States, at least two million people are infected with antibiotic-resistant bacteria and at least 23 thousand people die annually of these infections [8]. The World Health Organization (WHO) has dictated the control of antimicrobial resistance requiring a priority for the national government and health systems as one of the Global Strategy Recommendations [9]. With the unceasing emergence of new strains of global infectious pathogens in recent years, e.g., extensively antibiotic-resistant tuberculosis [10], avian influenza A (H5N1) and ebola [11], there is an urgent demand for exploring more efficient, broad-spectrum and long-lasting antimicrobial agents.

Conventional antimicrobial agents, which are prepared based on natural or low-molecular-weight compounds, are easily susceptible to resistance and may result in environmental contamination and toxicity to the human body due to biocidal diffusion [12,13]. In comparison, antimicrobial polymeric materials provide a valid approach addressing these problems by promoting antimicrobial efficacy and reducing residual toxicity [14,15]. In addition, antimicrobial polymers possess chemical stability and non-volatility, presenting long-term activity [16]. Different from the antimicrobial polymeric materials which are achieved by physically entrapping or coating organic and/or inorganic active agents to the materials during or after processing, polymers containing covalently bonded antimicrobial moieties avoid the problem of the permeation of low-molecular-weight biocides from the polymer matrices. Such antimicrobial polymers promise long-term durability in an environmentally friendly way [17,18]. Among them, the antimicrobial polymeric materials containing quaternary ammonium (QAS) and/or phosphonium salts (QPS) are probably the most widely used and studied antimicrobial polymers. Since Domagk discovered the antimicrobial property of benzalkonium chlorides in 1935 [19], generations of QAS with various structures have been explored as disinfectants. A survey on approximately 500 US EPA (Environmental Protection Agency) registered disinfectant products for households showed QAS are the most popular, being applied in 57.8% of the formulations [20]. The annual worldwide consumption of QAS was reported as 0.5 million tons in 2004, and was expected to exceed 0.7 million tons [21]. With structures and antimicrobial activities similar to QAS, QPS have therefore been developed presenting new progress in cationic biocides. Through either direct polymerization of monomers containing QAS/QPS groups or covalently incorporating QAS/QPS moieties within ordinary synthetic or natural polymers, polymeric QAS/QPS could achieve broad-spectrum antimicrobial activities due to the intrinsic property of the corresponding QAS/QPS [22,23,24]. Meanwhile, polymeric QAS/QPS contribute as potential drivers for conquering antibiotic-resistance [25,26,27,28,29,30].

The present review is focused on the well-established and newly developed antimicrobial polymeric materials with QAS/QPS moieties while discussing by sections the chemical structure and application, modes of antimicrobial action, factors affecting the antimicrobial activity, and the comparison of antimicrobial performance between polymeric QAS and polymeric QPS.

## 2. Polymeric Biocides with Pendant Quaternary Ammonium/Phosphonium Salts

One method of synthesizing polymers with pendant QAS/QPS is to prepare polymerizable QAS/QPS monomers which are subsequently polymerized or copolymerized with other monomers. Another method is the quaternization of polymers containing either tertiary ammonium/phosphonium groups or alkyl halides. In the direct polymerization process, the monomeric stability may be a limiting factor. In comparison, post-quaternization screens the potential disadvantage of monomeric stability, while the impact of neighboring groups and steric hindrance tend to limit the quaternization degree [31]. Since it is difficult to obtain complete functionalization by post-quaternization of polymeric tertiary ammonium/phosphonium salts, properties of the as-prepared polymers may vary in terms of the quaternization degree. For determining the antimicrobial efficiency of water-soluble QAS/QPS polymers, measurements of the minimum inhibitory concentration (MIC) and minimum bactericidal concentration (MBC) of polymers are the most common methods. The shaking flask test and inhibition zone measurement are two general approaches for evaluating the antimicrobial performance of water-insoluble polymers, in which the inhibition zone measurement is normally applied to detect the diffusion of biocidals.

### 2.1. Water-Soluble Quaternary Ammonium/Phosphonium Polymers

**Figure 1:**
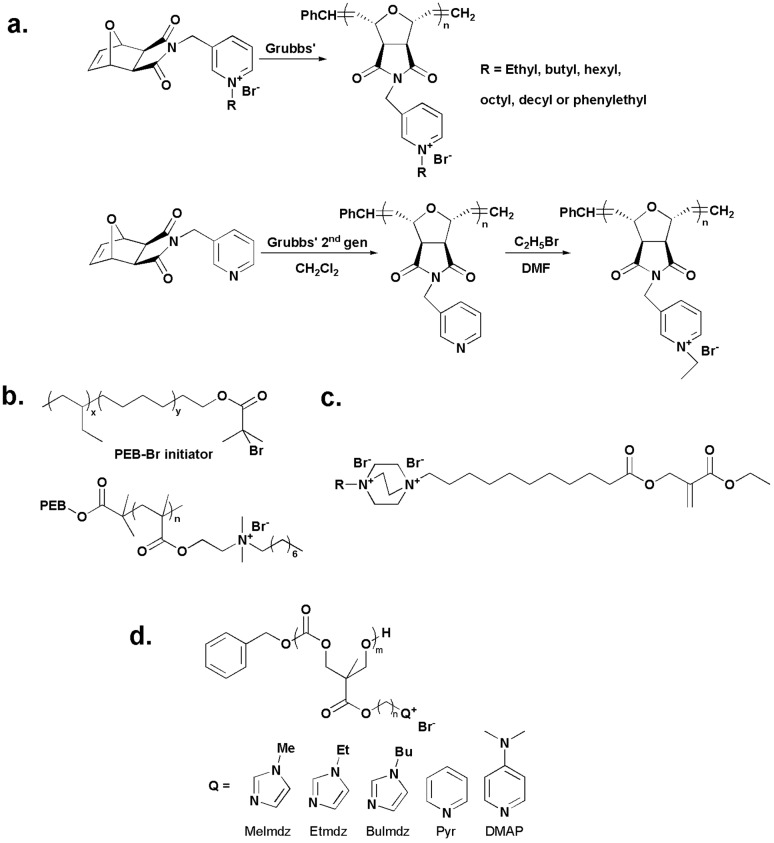
(**a**) Two synthetic pathways for polymers based on oxanorbornene derivatives, adapted with permission from [32]; (**b**) Octyl bromide quaternized poly(ethylene-*co*-butylene)-*b*-poly[2-(dimethylamino)ethyl-methacrylate] (PEB-*b*-PDMAEMA), adapted with permission from [33]; (**c**) Bis-quaternary ammonium methacrylate monomer based on 1,4-di-azabicyclo-[2.2.2]-octane, adapted with permission from [34]; (**d**) Antimicrobial polycarbonates with quaternized nitrogen-containing heterocycles, adapted with permission from [37].

To study the structure-activity relationship of quaternary pyridinium polymers, Eren *et al.* [32] synthesized a series of amphiphilic polynorbornenes with various quaternary alkyl pyridinium side chains (Figure 1a). The preparation of pyridinium functionalized polynorbornene with an ethyl pendant group was conducted using two different methods, *i.e.*, direct-polymerization and post-quaternization, and polynorbornenes with 100% and 85% of quaternization degrees were obtained, respectively. By evaluation of their antibacterial activity against *Escherichia coli* (*E. coli*) and *Bacillus subtilis* (*B. subtilis*) and hemolytic activity against fresh human red blood cells, it was found that the MICs of both samples were 200 µg/mL while the latter one was twice as hemolytic as the former one. It implied that the synthetic route of polymeric QAS may impact their biological activity due to the effect of the quaternization degree on the hydrophobic/hydrophilic balance of the polymers.

Lenoir *et al.* [33] synthesized an antimicrobial surfactant via quaternization of the amino groups of poly(ethylene-*co*-butylene)-*b*-poly[2-(dimethylamino)ethyl-methacrylate] (PEB-*b*-PDMAEMA) copolymer with octyl bromide. The block copolymers were prepared by bromide-capped PEB initiated atom transfer radical polymerization (ATRP) of DMAEMA (Figure 1b). The shaking flask test against *E. coli* demonstrated the prepared surfactant had antimicrobial activity comparable to that of a commonly used disinfectant, *i.e.*, benzalkonium chloride.

It has been proven that a long alkyl chain substituent, *i.e.*, at least eight carbons, renders QAS highly antimicrobial [34]. Considering this, Dizman *et al.* [35] synthesized a methacrylate monomer containing pendant QAS based on 1,4-di-azabicyclo-[2.2.2]-octane, which contained either a butyl or a hexyl group (Figure 1c). Although the monomers did not show any antimicrobial properties, the corresponding homopolymers were effectively bactericidal against *Staphylococcus aureus* (*S. aureus*) and *E. coli*. And their activity was found to be dependent on the length of the hydrophobic segment, *i.e.*, the polymer with hexyl groups was more effective than the one with butyl groups.

Nonaka and coworkers [36] prepared antimicrobial QPS-pendant polymers with thermosensitivity by copolymerization of *N*-isopropylacrylamide with methacryloyloxyethyl trialkyl phosphonium chloride. The copolymers with octyl groups in QPS showed a lower LCST (lower critical solution temperature) and higher antimicrobial activity compared to those with either ethyl or butyl groups.

To investigate the antimicrobial activity of biodegradable cationic polycarbonates with quaternized nitrogen-containing heterocycles, Yang *et al.* [37] synthesized a series of polycarbonates with propyl and hexyl side chains followed by quaternization with different *N*-heterocycles (Figure 1d). All the *N*-heterocycle quaternized polycarbonates exhibited higher antimicrobial efficiency against bacteria and fungus compared to their trimethylamine quaternized analogues. The amphiphilicity of the polymers was found to be an important factor affecting their antimicrobial performance and hemolytic activity. Compared to polymers containing *n*-PrBr side chains, polymers containing *n*-HexBr side chains showed higher antimicrobial activity against various strains of bacteria and fungus, as well as higher hemolytic activity toward mammalian red blood cells.

The cellular membranes of most bacteria are negatively charged and have proven to be the target site of cationic biocides [38,39,40]. The antibacterial mechanism of biocidal QAS/QPS, a class of membrane-active cationic biocides, has been proposed to be penetration into the cell wall and destructive interaction with the cytoplasmic membrane, followed by the leakage of intracellular components and consequent cell death [41,42]. Compared to low-molecular-weight QAS/QPS, polymeric QAS/QPS have higher positive charge density which promotes initial adsorption onto the negatively charged bacterial surfaces and disruption of cellular membranes, resulting in significantly enhanced antibacterial activity [43,44]. Benefiting from the rapid development of characterization technology, various advanced technologies, including AFM [45,46], fluorescence correlation spectroscopy [47,48], and/or tracking the leakage of cellular constituents [49], have been applied to investigate the action mode of antimicrobial materials. These studies provide intuitive and persuasive evidence for supporting the hypothesis about the antimicrobial mechanism of cationic biocides. At the molecular level, a model lipid bilayer membrane has been employed to mimic the permeability barrier of cellular membrane for understanding the interaction between cationic biocides and bacterial membrane [50,51]. The electrostatic interactions between the cationic polymers and the lipid headgroups result in the formation of interfacial complexes within the outer leaflet. The interaction also induces flip-flop of anionic lipid molecules from the inside to the outside leaflet, followed by significant distortions and phase separation of the phospholipid bilayer [52,53].

It is worth noting that the structures of the cellular envelope are different between Gram-positive and Gram-negative bacteria. Gram-positive bacteria have a loosely packed polyglycane cell wall, facilitating the penetration of antimicrobial polymers through it and interaction with the membrane, while Gram-negative bacteria have an additional membrane composed of a phospholipid bilayer, which acts as a barrier against the polymeric biocides [54,55,56]. As a whole, Gram-positive bacteria exhibit lower resistance to biocides compared to Gram-negative ones [57,58,59,60].

### 2.2. Water-Insoluble Quaternary Ammonium/Phosphonium Polymers

**Figure 2:**
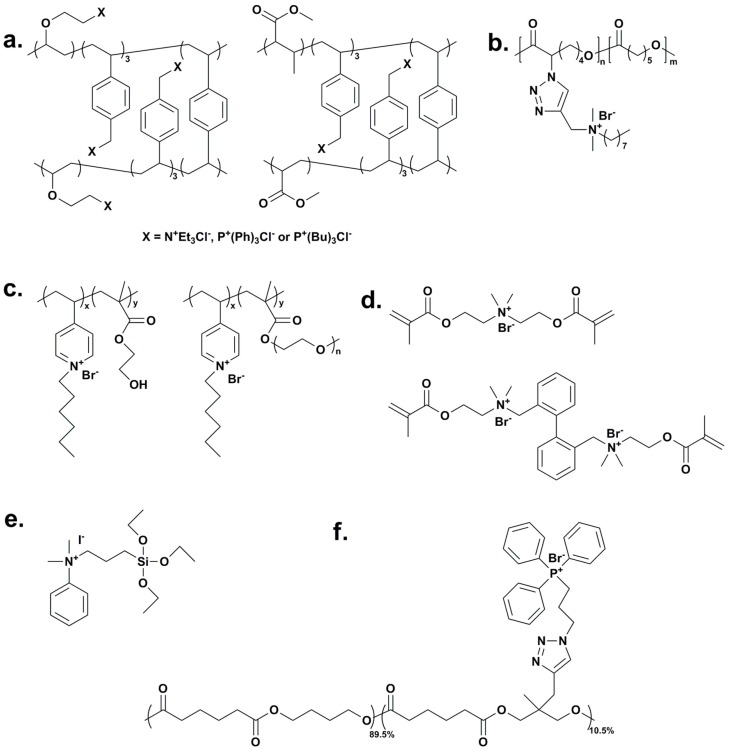
(**a**) Immobilization of quaternary ammonium (QAS) or phosphonium salts (QPS) onto crosslinked copolymers, adapted with permission from [61]; (**b**) Antimicrobial poly(*ɛ*-caprolactone), adapted with permission from [62]; (**c**) Quaternized poly(VP-*co*-HEMA) and poly(VP-*co*-PEGMA), adapted with permission from [63]; (**d**) Two types of ionic dimethacrylate monomers containing QAS for preparing antimicrobial dental materials, adapted with permission from [64]; (**e**) Triethoxysilane terminated QAS, adapted with permission from [65]; (**f**) QPS functionalized alkyne-containing poly(butylene adipate), adapted with permission from [66].

Kenawy and coworkers [61] developed two kinds of crosslinked copolymers through copolymerization of vinylbenzyl chloride with 2-chloroethyl vinyl ether or methylmethacrylate using divinylbenzyl chloride as the crosslinker, followed by quaternization with tertiary amines/phosphines (Figure 2a). The antimicrobial properties of prepared copolymers were studied using a cut plug method against bacteria and fungi. By calculating the surviving ratio of microbes, all the tested copolymers exhibited good antimicrobial performance, among which, the crosslinked polymer quaternized with triphenylphosphonium salt was the most effective against the tested bacteria and fungi.

Biodegradable poly(*ɛ*-caprolactone) (PCL) with antimicrobial property [62] was prepared by grafting alkyne-containing QAS to pre-synthesized azide-containing PCL (Figure 2b). Accompanying the biodegradability, a biocidal effect of the QAS-modified PCL was observed, which was analyzed via the shaking flask test against *E. coli*.

To improve the hydrophilicity and biocompatibility of quaternized poly(vinylpyridine) (PVP), high hydrophilic and biocompatible monomers hydroxythylmethacrylate (HEMA) and polyethylene glycol methyl ether methacrylate (PEGMA) were incorporated via copolymerization with 4-vinyl pyridine, respectively [63]. The pyridine groups were quaternized with hexylbromide, resulting in cationic copolymers with different compositions (Figure 2c). By recording the photoluminescence attenuation induced by *E. coli* cells in contact with the polymer coated glass slides, optimally formulated copolymers were found to be over 20 times more active than the quaternized homo-PVP. Combined with the results of the contact angle test, it was concluded that the enhancement of hydrophilicity could significantly improve both the antimicrobial property and biocompatibility of polymeric materials.

To develop novel antimicrobial dental materials, two types of ionic dimethacrylate monomers containing QAS (Figure 2d) were synthesized via the Menschutkin reaction, and one of them was incorporated into a bisphenol A glycerolate dimethacrylate (BisGMA): triethylene glycol dimethacrylate (TEGDMA) (1:1) resin by photopolymerization [64]. The antimicrobial test and macrophage viability assay indicated that the incorporation of the cationic monomer as low as 10 mol % rendered the resin effectively antimicrobial and highly biocompatible.

Marini *et al.* [65] prepared antimicrobial hybrid coatings containing a novel trialkoxysilane QAS (Figure 2e) covalently bonded to the organic-inorganic network using a sol-gel process. The antimicrobial performance of PE films with the QAS-containing coatings was evaluated against *E. coli* and *S. aureus* at different contact times. Results showed the film, which went through repeated washings, maintained excellent antimicrobial property, *i.e.*, about 99% of biocidal efficiency, even after 96 h.

A novel alkyne-containing poly(butylene adipate) (Figure 2f) was developed and functionalized with QPS via a copper-catalyzed azide-alkyne “click” reaction [66]. The antimicrobial activity of the functionalized polyester was studied against *E. coli*, presenting significant reduction of cell counts both in dispersion and on the surface. The QPS functionalized polyester exhibited great potential application as an antimicrobial packaging film for food.

Various polymeric QAS/QPS, which possess highly antimicrobial activities in solution, exhibit significantly decreased antimicrobial efficiency after being crosslinked or insolubilized. While Tiller *et al.* [67] revealed that the antimicrobial activity of water-insoluble polycations can be preserved as long as the polymeric chains are long and flexible for penetration through the bacterial membranes. Bieser and Tiller [68] prepared a series of water-insoluble *N*-alkyl-*N*,*N*-dimethyldeoxyammonium celluloses, and found the celluloses modified by *N*,*N*-dimethyldodecyl ammonium exhibited antimicrobial properties while those modified by *N*,*N*-dimethylbutyl ammonium did not. Based on the findings, they proposed a “phospholipid sponge effect” to explain the antimicrobial mechanism of water-insoluble polycations, *i.e.*, the biocidal action is triggered by the interaction between the negatively charged phospholipids in the cellular membranes and the positively charged surfaces.

## 3. Polymers with Quaternary Ammonium/Phosphonium Salt within the Main Chain

Cationic polymers containing positive nitrogens/phosphors in the backbone, known as ionene polymers, also possess antimicrobial properties due to the biocidal QAS/QPS within the main chain [69,70,71]. Ionene polymers are typically prepared either by step-growth polymerization of suitable monomers (e.g., the Menshutkin reaction between alkyl dihalides and nucleophilic ditertiary amines, self-polyaddition of aminoalkylhalides) or cationic functionalization of precursor polymers [72,73,74].

Through facile condensation polymerization of benzyl amine and epichlorhydrin, polyelectrolytes with QAS in the main chain were synthesized [75]. The results of the agar well diffusion test showed that the ionene polymers had antimicrobial properties against bacteria, yeast and fungi, among which their antibacterial and anti-yeast activities were dependent on the chain length.

**Figure 3:**
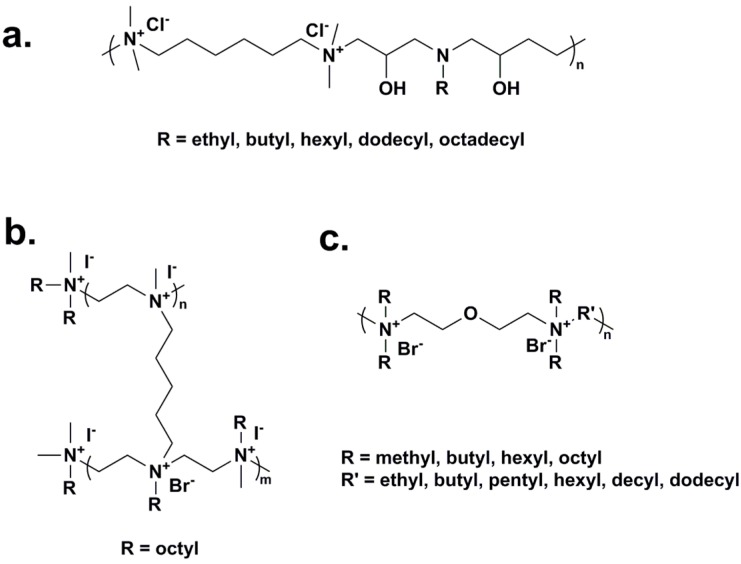
(**a**) Comb-like ionenes with aliphatic side chains, adapted from [76]; (**b**) Polyethyleneimine (PEI)-based ionenes for preparation of dental composites, adapted with permission from [78]; (**c**) Alkyloxyethylammonium ionenes, adapted with permission from [86].

A series of comb-like ionenes (Figure 3a) were synthesized for the preparation of antimicrobial and antistatic polyethylene [76,77]. Compared with linear ionenes, the comb-like ionenes with long aliphatic side chains presented a higher and faster biocidal effect against *E. coli*. In addition, the comb-like ionenes showed antimold properties against *Aspergillus niger* (*A. niger*) and *Chaetomium globosum* (*C. globosum*). Blending the prepared ionenes with low density polyethylenes (LDPE) resulted in functional PE sheets possessing both antimicrobial and antistatic properties.

Beyth and coworkers [78,79] reported the synthesis of alkylated polyethyleneimine (PEI)-based nanoparticles containing QAS antimicrobial groups (Figure 3b). The cationic nanoparticles were synthesized from crosslinked PEI, followed by quaternization with bromooctane and methylation with methyl iodide. By incorporating the nanoparticles at a concentration as low as 1% into commercial dental resin composites during polymerization, dental composites with strong antimicrobial activity against *Streptococcus mutans* (*S. mutans*) were achieved. Over one month, the modified resin composites maintained full activity without leaching of nanoparticles and mechanical properties. In addition, XTT (2,3-bis(2-methoxy-4-nitro-5-sulfophenyl)-5-[(phenylamino)carbonyl]-2H-tetrazolium hydroxide) assay and cytokine analysis proved that the modified resin composites did not change either the viability or activity of the macrophage as compared to the native composites, indicating the antimicrobial dental resin composites possessed high biocompatibility for potential application *in vivo* [80].

Similar to the antimicrobial polymers with pendant QAS/QPS, the biocidal activity of ionene polymers arises from the cationic moieties. Rembaum’s report [81,82] revealed the bactericidal mode of ionenes was to form complexes with heparin and DNA, accompanied by adhesion, aggregation and lysis of bacterial cells. Ikeda *et al.* [83] explored the interaction between ionene polymers and phospholipid bilayer membranes. Compared to those with flexible spacers, the ionenes with rigid spacers exhibited stronger interaction with phospholipid bilayers, resulting in phase separation of bilayer membranes. Also incorporating hydrophilic moieties into the spacers induced the loss of ability to initiate phase separation. Narita and coworkers [84,85] studied the effect of charge density and hydrophobicity of ionene polymers on yeast protoplast disruption. The ionenes containing separated longer hydrophobic segments but with lower charge densities exhibited more effectively biocidal ability than those with higher charge densities, suggesting that the hydrophobicity is the dominant factor for cell disruption. Mattheis *et al.* [86] synthesized various alkyloxyethylammonium ionenes with different alkyl chain substituents on the nitrogens and aliphatic spacers (Figure 3c) via step-polymerization of alkyl dibromides with bis(2-*N*,*N*-dialkylamino)ethyl ethers. The effects of counter ion, alkyl spacer, and length of the pendant alkyl chains on the antimicrobial performance were investigated via broth dilution method. Generally, appropriate pendant substituents (*i.e.*, short methyl or relatively long octyl groups) and long backbone alkyl spacer endowed ionenes with high antimicrobial activities. Nevertheless, the counter anions, among the investigated ionenes containing bromide, hydroxide and phosphate, played a minor role in their biocidal performance.

## 4. Hyper-Branched and Dendritic Polymers

Branched polymers, including star-shaped, dendritic and hyper-branched architectures, have attracted attention as potential antimicrobial agents. These three-dimensional polymers provide an alternative way for design and preparation of novel antimicrobials, due to the compact structure and multiple functionality [87,88,89]. Dendritic polycations are typically synthesized by multi-step procedures, *i.e.*, chain growth, step growth and/or living chain growth, in either a divergent or convergent method, while hyper-branched polycations can be prepared by single-step reactions, *i.e.*, polycondensation, polyaddition, and/or a ring opening reaction [90,91,92].

**Figure 4:**
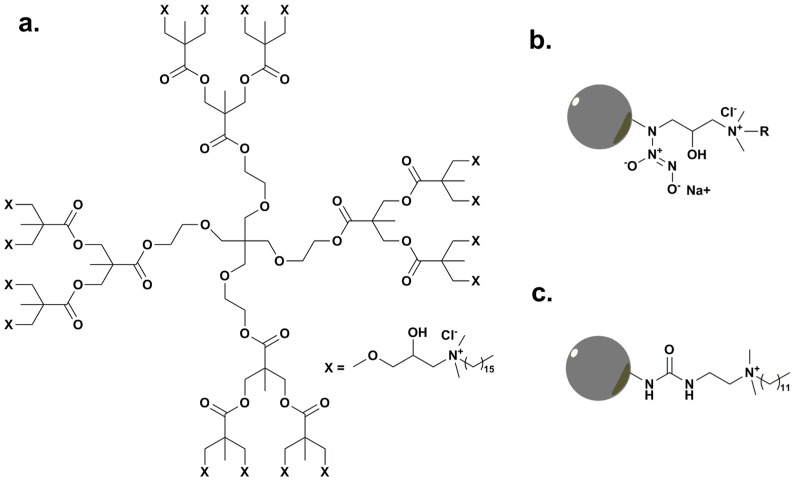
(**a**) QAS modified hyper-branched polyester, adapted from [93]; (**b**) QAS-modified poly(amidoamine) dendrimers with NO-releasing function, adapted with permission from [95]; (**c**) QAS-modified poly(propylene imine) dendrimers, adapted with permission from [97].

A QAS functionalized hyper-branched polyester (Figure 4a) was developed by modification of branched polyester Boltorn H20 with hexadecyldimethylamine using epichlorohydrin as the linker [93]. The prepared hyper-branched polyester was employed to modify silk fabric and the antimicrobial activity of the fabric after antibacterial finishing was evaluated against *E. coli* and *S. aureus*. The antimicrobial hyper-branched polyester treated fabric showed excellent antimicrobial properties even after washing 15 times.

Asri *et al.* [94] covalently tethered QAS onto the surface of hyper-branched polyurea coatings and studied their contact-antimicrobial activity. Through culture-based assay, confocal laser scanning microscopic examination, and AFM experiments, it was found that hyper-branched QAS coatings possessed great contact-killing activities towards adhered bacteria without leaching of bactericidal.

Worley *et al.* [95] reported the synthesis of nitric oxide (NO)-releasing QAS-functionalized poly(amidoamine) (PAMAM) dendrimers. The dendrimers were modified with QAS containing different lengths of alkyl chains, followed by modification of secondary amines with *N*-diazeniumdiolate (Figure 4b). The antimicrobial activity of the dual-action (*i.e.*, NO-releasing and modified QAS) PAMAM was found to be dependent on the dendrimer generation and alkyl chain length of QAS. Longer QAS alkyl chains, *i.e.*, octyl and dodecyl, rendered PAMAM higher bactericidal than shorter chains, *i.e.*, methyl and butyl, for both G1 and G4 dendrimers, while the additional function of NO release significantly improved the antimicrobial activity of PAMAM with shorter QAS alkyl chains instead of those with longer alkyl chains.

Chen *et al.* [96,97] and Charles *et al.* [98] successfully functionalized poly(propylene imine) (Figure 4c) and poly(amidoamine) dendrimers with dimethyl dodecyl ammonium groups respectively, and investigated their antimicrobial properties. It was found that dendrimers containing 16 QAS groups per macromolecule exhibited two orders of magnitude higher bactericidal efficiency against Gram-negative bacteria compared to their mono-functional counterparts [99]. A comparison of low-molecular-weight, polymeric and dendritic biocides in their antimicrobial activity at each step is summarized in Table 1 [100].

**Table 1:** Comparison of molecular biocides in their interaction with bacteria.

Step	Low-Molecular-Weight Biocides	Polymeric Biocides	Dendritic Biocides
Initial adsorption	Low	High	High
Diffusion past the cell wall	High	Low	Medium
Binding to the membrane	Low	Medium	High
Disruption of the membrane	Low	Medium	High

## 5. Immobilization of QAS/QPS on Material Surfaces

Antimicrobial modification of material surfaces is an alternative way of preventing the formation of highly resistant biofilms, and can be achieved by various methods [101,102,103,104,105,106,107,108]. Among them, covalently attaching biocidal QAS/QPS and/or corresponding polymers onto a material surface is an effective approach for rendering surfaces permanent contact-active antimicrobial [109,110,111,112,113,114,115]. In order to prepare permanent antimicrobial surfaces, mainly three elaborate techniques have been developed, *i.e.*, a surface grafting method, plasma polymerization and layer-by-layer (LbL) deposition [116,117,118,119,120,121].

Poly(4-vinyl-*N*-alkylpyridium bromide) with various alkyl chain lengths, *i.e.*, from propyl to hexadecyl, was covalently attached onto amino-modified glass slides [67]. By comparison, the glass slide immobilized with hexyl-PVP was found to be the most effective in decreasing the bacterial cell counts, while neither the decyl-PVP nor non-alkylated PVP modified glass slides showed antimicrobial properties.

Various polymeric QAS modified woven textiles have been developed by covalently bonding alkylated PEI onto the textile surfaces [122]. The results of the antimicrobial test demonstrated that the immobilization of polymeric QAS rendered wool, cotton, nylon and polyester not only effectively antibacterial but also antifungal.

**Figure 5:**
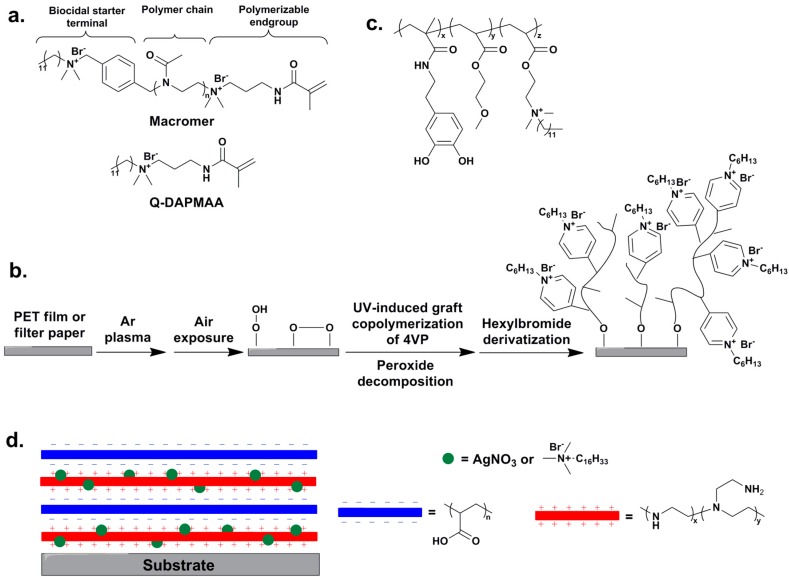
(**a**) Acrylate-based polymer containing one biocidal QAS and one polymerizable end group, adapted with permission from [123]; (**b**) Surface functionalization for conferring antimicrobial properties to polymeric and cellulosic surfaces, adapted with permission from [124]; (**c**) Antimicrobial amphiphilic polycations with catechol functional group, adapted with permission from [126]; (**d**) Polyelectrolyte multilayer films containing cetrimide and silver, adapted with permission from [131].

Waschinski *et al.* [123] designed and prepared a novel acrylate-based material with contact-active antimicrobial property via UV-induced radical copolymerization of biocidal macromers with HEMA and 1,3-glyceroldimethacrylate on methacrylate modified glass slides. The biocidal macromers were composed of biocidal QAS terminal groups, a poly(2-methyl-1,3-oxazoline) chain with various spacer lengths and methacrylamide polymerizable groups (Figure 5a). In contrast to the films made from the comonomer without the polymeric spacer (*N*-[3-(methacryloylamino)propyl]-*N*,*N*-dimethyldodecylammonium bromide, Q-DAPMAA), which presented inhibition zones after a two-day washing time while losing activity after four days, all the films containing the biocidal macromers had no observable inhibition zones and preserved highly antimicrobial activity even after 45 days of washing.

Cen *et al.* [124] immobilized antimicrobial QAS onto the surfaces of PET films and filter papers by grafting copolymerization of 4-vinylpyridine and subsequent quaternization of the grafted pyridine groups with hexyl bromide (Figure 5b). The results of both the waterborne and airborne assay against *E. coli* demonstrated that both the PET films and filter papers were conferred highly bactericidal properties after being surface-modified by QAS.

Jamapala *et al.* [125] reported a novel bottom-up synthetic process for preparing antimicrobial surfaces. Firstly, the surfaces of stainless steel, treated by O2 and hexamethyldisiloxane plasma, and cellulose-based filter paper were functionalized with secondary amines via ethylene diamine plasma treatment. Afterwards, the plasma-deposited amines reacted with hexyl bromide and subsequently, QAS-immobilized surfaces were formed by quaternization of the tertiary amines with methyl iodide. The bactericidal properties of the modified surfaces were evaluated against *S. aureus* and *Klebsiella pneumoniae* (*K. pneumonia*), showing that the immobilization of QAS rendered both stainless steel and filter paper surfaces bactericidal with non-leaching of biocidal.

Recently, a new and simple dip-coating strategy using catechols as the anchoring reagents was developed for preparing permanently antimicrobial surfaces [126]. Tripolymers composed of different molar ratios of catechol moieties, methoxyethyl groups and QAS with long alkyl chains were synthesized (Figure 5c) and coated onto glass slides without surface pretreatment. The incorporation of the biocidal QAS and the hydrophilic comonomers, which was employed to promote the interaction between polymers and bacterial cells by tuning the amphiphilic balance, endowed these coatings with great bactericidal properties against both Gram-positive and Gram-negative bacteria on contact. Contrary to the control coatings without catechol groups, the coatings containing catechols prevented the development of biofilms for up to 96 h, and did not show leaching of the biocidal. It demonstrated that the catechol groups significantly enhanced the immobilization of polymers onto surfaces due to the formation of hydrogen bonds, covalent bonds, and/or strong physical interactions [127,128,129].

Various antimicrobial surfaces have been developed by combining covalently bonded QAS/QPS with releasable nanoparticles [130], metal ions and/or clays [131], which results in dual-functionalized antimicrobial properties. Grunlan [132] and Li [133] developed antimicrobial multilayer films containing both QAS and silver ions by employing the LbL method, respectively. The polyelectrolyte multilayer films reported by Grunlan *et al.* were prepared by alternately dipping a poly(ethylene terephthalate) (PET) substrate into solutions of biocidal agents (*i.e.*, cetyltrimethylammonium bromide (CTAB) and/or silver) containing PEI and poly(acrylic acid) (Figure 5d). Inhibition zone measurement against *S. aureus* and *E. coli* indicated that the films made with CTAB had higher antimicrobial activity compared with the films containing either silver alone or both CTAB and silver. The antimicrobial thin film coatings designed by Li *et al.* were composed of two distinct functional layers, *i.e.*, a reservoir for loading and releasing of silver ions and a nano-particle surface cap immobilized with [3-(trimethoxysilyl)propyl]octadecyl-dimethylammonium chloride. The dual-functional coatings bearing both biocidal-releasing and contact bacterial killing properties exhibited great initial bactericidal efficiency, and simultaneously retained antimicrobial activity even after the silver depletion.

Modification by polymeric QAS/QPS can render material surfaces antimicrobial against either waterborne or airborne bacteria on contact [134,135,136,137]. One hypothetical mechanism has been raised for the explanation of their antimicrobial action. Similar to their action mode in solution, the polycations immobilized on material surfaces penetrate and disrupt the bacterial wall/membrane via electrostatic interaction with the negatively charged phospholipids within the cellular membrane [138,139,140,141,142]. An ion exchange between the mobile cations within the bacterial membrane and the positively charged surfaces may be induced during the bactericidal process [143,144,145].

## 6. Effect of Counter Anion and Amphiphilic Balance on the Antimicrobial Activity

The characteristics of the polymeric QAS/QPS such as molecular weight, charge distribution and density, nature of counter anion, amphiphilic balance, *etc*., affect their antimicrobial properties. In this section, the effect of counter anion and hydrophobicity/hydrophilicity are discussed in detail.

### 6.1. Counter Anion

As mentioned before, biocidal polycations inactivate bacteria by disrupting cellular membrane due to electrostatic interaction with negatively charged bacteria. Replacement of Ca^2+^ and/or Mg^2+^ on cellular membranes by biocidal cations may occur during the sterilization process [146,147]. In this regard, the identity of the counter anion plays a key role in the antimicrobial performance of polymeric QAS/QPS.

**Figure 6:**
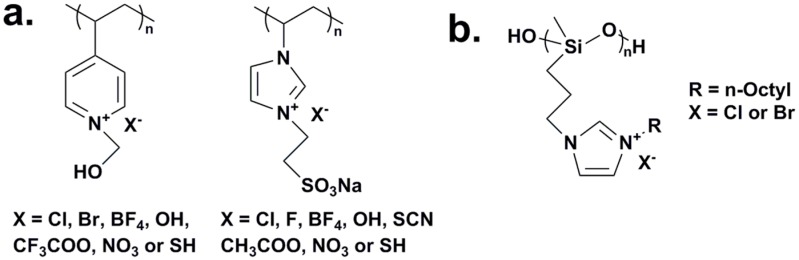
(**a**) Quaternary poly(4-vinyl-2-hydroxyethyl pyridinium) chloride and poly[1-vinyl-3-(2-sulfoethyl imidazolium betaine)] with various counter anions, adapted from [148,149]; (**b**) [*N*-3(*N*'-*n*-octylimidazolio)propyl]methyl siloxane halide polymers, adapted with permission from [152].

For tuning the antimicrobial activity of polycations by exchanging counter anions, Chauhan’s group prepared two types of bioactive polymers, *i.e.*, poly(4-vinyl-2-hydroxyethyl pyridinium) chloride [148] and poly[1-vinyl-3-(2-sulfoethyl imidazolium betaine)] [149], and replaced the chloride and bromide counter anions with various anions via an anion exchange reaction (Figure 6a), respectively. Specifically, Cl^−^ of the original pyridinium polymer was exchanged with Br^−^, OH^−^, SH^−^, NO_3_^−^, BF_4_^−^ and CF_3_COO^−^ and their antimicrobial properties were studied against fungi (*A. niger* and *Mucor circenelliods* (*M. circenelliods*)) and bacteria (*Bacillus coagulans* (*B. coagulans*) *BTS-3*). In comparison, the polymer with OH^−^ as the counter anion presented the strongest antimicrobial activity with MIC values of 520 and 1040 ppm against *A. niger* and *M. circenelliods* fungi, respectively, and 65 ppm against *B. coagulans* bacterium. Br^−^ from the original polysulfobetaine (PSB) was replaced with Cl^−^, F^−^, OH^−^, SH^−^, SCN^−^, NO_3_^−^, BF_4_^−^ and CH_3_COO^−^ and the antimicrobial activities of prepared polysulfobetaines were determined against three fungi and two bacteria. For different types of microorganism, PSBs with different counter anions exhibited significant differences in term of antimicrobial activity. For Gram-positive bacteria, *i.e.*, *B. coagulans*, [PSB]^+^OH^−^ possessed the strongest activity. In contrast, for Gram-negative bacteria, *i.e.*, *Pseudomonas aeruginosa* (*P. aeruginosa*), [PSB]^+^F^−^, [PSB]^+^SH^−^ and [PSB]^+^NO_3_^−^ were the most effective ones. Regarding antifungal activity, [PSB]^+^SH^−^ showed maximum activity against *M. circenelliods* while [PSB]^+^OH^−^ was most effective against *Byssochlamys fulva* (*B. fulva*). It was suggested that the structure of the counter anion has a profound effect on the efficiency and selectivity towards different microbes due to the discrepancy of polymer morphology and the solubility of polycations in water, resulting in various degrees of antimicrobial performance.

Kanazawa *et al.* [150] developed a series of tributyl(4-vinylbenzyl)phosphonium salts with different counter anions and corresponding polymers. By comparing the antimicrobial activity of polymers against *S. aureus*, it was found that the structure of the counter anion strongly affected their antimicrobial performance, resulting in the activity in the order of Cl^−^ > BF_4_^−^ > ClO_4_^−^ > PF_6_^−^. The trend was in accordance with the order of *Ksp* (solubility product constant) of studied phosphonium salts. It was postulated that the high antimicrobial activity of polymeric QPS with Cl^−^ was due to facilitating ionic dissociation of QPS to free ions, while the activity was decreased for polymers with the counter anions forming tight ion-pairs with the phosphonium ions.

In studying the effect of halogen ions on the bactericidal property of polycations, Panarin *et al.* [151] homopolymerized vinyl amine and methyl acrylate with pendent QAS and evaluated their antimicrobial activities. No difference in the antimicrobial behavior was shown among the polymeric QAS with counter anions of chloride, bromide, and iodide. Similarly, the cationic biocidal polysiloxanes with pendant imidazolium salt (Figure 6b) did not exhibit different antimicrobial activities towards both Gram-negative and Gram-positive bacteria when different counter anions, chloride or bromide, were used [152]. Nevertheless, a series of quaternary ammonium functionalized poly(propyleneimine) dendrimers developed by Chen *et al.* [94] exhibited dependence of antimicrobial activity on the halogen counter anion. By evaluation of their antimicrobial properties against *E. coli* and *S. aureus* via a bioluminescence method, the dendrimer biocide with Br^−^ was more potent than that with Cl^−^. The difference in antimicrobial activity between the dendrimers with chloride and bromide counter anions was not expected since both ions were able to dissociate freely in water. Xie *et al.* [153] also found the same potency difference in investigating the antimicrobial functions of a glass-ionomer cement. Novel polymeric QAS-containing polyacids with different chain lengths and counter anions were applied to formulate the cements and their antimicrobial performance was studied against *S. mutans*. With the same chain length, the cements containing QAS bromide possessed significantly higher bactericidal efficiency than those containing QAS chloride, although their compressive strength values were not statistically different from each other. A systematic research with emphasis on the role of the counter anion in the antibacterial properties of QAS was reported by Priefer’s group [154]. By monitoring the Inhibition Zone of tetrabutylammonium (TBA), over thirty types of anions including halogen ions were studied. Based on the result, it was proposed that if the counter anion bound strongly to the TBA cation, it would be difficult for dissociation, and the displacement of Ca^2+^ and Mg^2+^ during their antibacterial action would be hampered as a result. This may explain the different antimicrobial activities of polymeric QAS/QPS with different halogen ions.

### 6.2. Hydrophobicity-Hydrophilicity Balance

It has been well acknowledged that the hydrophobicity-hydrophilicity balance, also referred to as “amphiphilic balance”, is a key factor in the antimicrobial activity of polymeric QAS/QPS [155,156,157]. More specifically, the hydrophobicity-hydrophilicity balance is dependent on the length of the substituted alkyl chain [158,159,160], the alkyl spacer between neighboring ammonium groups [161], hydrophobicity/hydrophilicity, and the balance of cationic moieties and hydrophobic groups [162,163,164,165], impacting both the antimicrobial activity and biocompatibility of polycations [166,167,168]. Variation in the amphiphilic balance leads to different affinities between the polycations and the bacterial membranes. Hydrophobic allyl chains facilitate polymeric QAS/QPS binding and diffusing through cellular membranes, while excessive hydrophobicity tends to block membrane penetration and increase cytotoxicity.

Novel methacrylamide polymers possessing both antimicrobial activity and thermo-sensitivity were developed by copolymerizing pre-synthesized pyridine-pendant methacrylamide with *N*-isopropyl acrylamide and quaternizing the pyridine groups using bromoalkanes containing different lengths of alkyl chains [169]. Both the thermo-sensitivities and antimicrobial activities were found to be dependent on the alkyl chain length, e.g., the polymeric QAS containing a 14 carbon alkyl chain exhibited the highest activity compared to those with 12 and 16 carbon alkyl chains.

**Figure 7:**
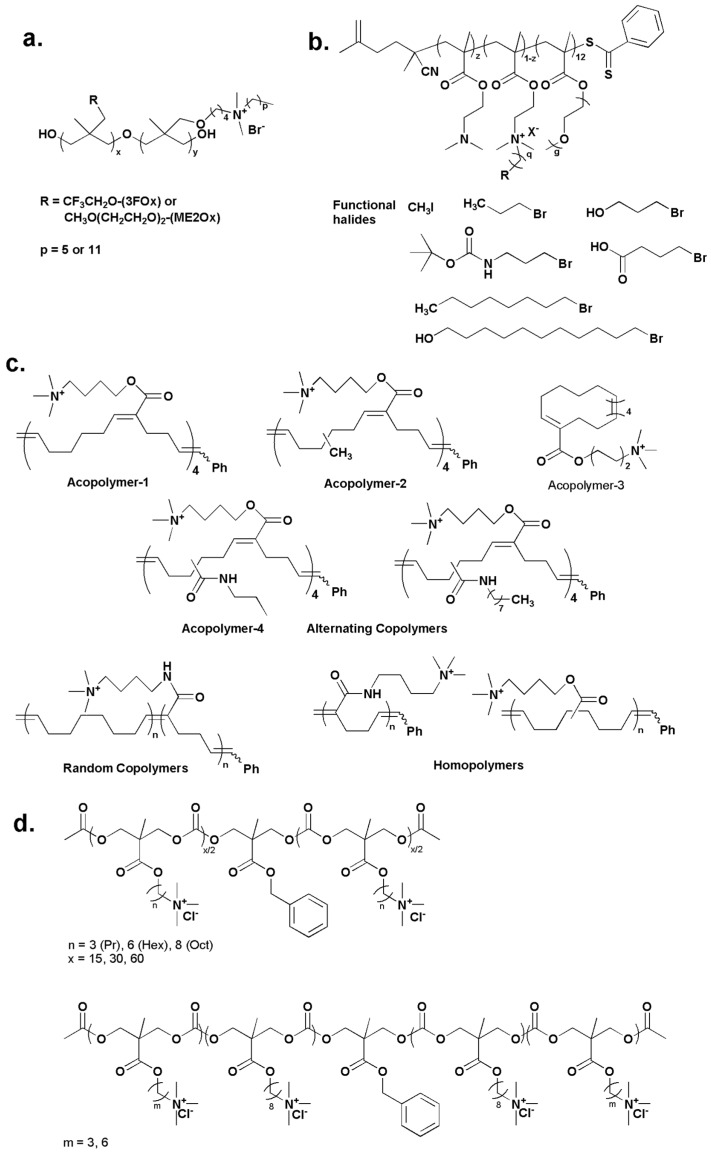
(**a**) Random copolymeric oxetane with QAS and either trifluoroethoxy or PEGlyted side chains, adapted with permission from [170]. (**b**) Well-defined pegylated-polymers with different amphiphilic balance and chemical functionalities, adapted with permission from [171]; (**c**) Synthetic antimicrobial peptides with alternating, random and uniform backbones, adapted with permission from [172]. (**d**) Antimicrobial polycarbonates with different amphiphilic balance, adapted with permission from [173].

Contact antimicrobial polyurethane surfaces containing soft block side chains [170] were developed by random-copolymerization of 1,3-propylene oxide with QAS and of either polyethylene glycol (PEG) or trifluoroethyoxy side chains (Figure 7a). The antimicrobial tests against *S. aureus*, *E. coli* and *P. aeruginosa* showed that both the PEG- and trifluoroethyoxy-containing polyurethane coatings modified by QAS with six carbon chains were more effectively bactericidal compared to those modified by QAS with 12 carbon chains.

Venkataraman *et al.* [171] designed and prepared well-defined pegylated-polymers via reversible addition-fragmentation chain transfer (RAFT) polymerization of commercially available monomers, *i.e.*, 2-(dimethylamino)ethyl methacrylate and oligo(ethylene glycol) methyl ether methacrylate. By employing an efficient post-quaternization strategy, the tertiary amines were quaternized with various functional halides resuling in polymeric QAS with different amphiphilic balance and chemical functionalities (Figure 7b). Among the series of polymers, of which the antimicrobial activities were evaluated against *B. subtilis*, the polymers containing QAS with shorter alkyl spacers exhibited higher activity. In agreement with Eren’s study mentioned in 2.1., the amphiphilic balance was found to affect the hemolysis of polymers. Compared to the pegylated-polymers consisting of short alkyl spacer, similar polymers with longer alkyl spacer were highly hemolytic due to the long alkyl groups facilitating diffusion of the polymeric QAS into the blood cell membrane and causing cell lysis as a result.

A series of polymeric QAS, in which the QAS side chains were scattered within unsubstituted backbone or with lipophilic side chains (Figure 7c), were synthesized to mimic antimicrobial peptides (AMP) [172]. The lipophilicity of the polymers was tuned by exchanging the substituents on the cyclohexene and/or cyclobutene units. By comparing the MIC values of the alternating copolymers with different hydrophobic substituents, *i.e.*, Acopolymer-1, -2, -3 and -4, it was found that the increase of hydrophobicity did not enhance the antimicrobial efficiency of the synthetic AMPs. In addition, the hydrophobic spacer between adjacent ammonium moieties along the backbone must be at least 8~10 Å to achieve effective antimicrobial properties.

Engler *et al.* [173] studied the effect of amphiphilicity of a series of homopolymer polycarbonates (Figure 7d) on their antimicrobial activity and selectivity toward microbes over mammalian cells. The amphiphilic balance was tuned by varying the spacer between the cationic moiety and the polymer backbone. By comparing the MIC values of homopolymers with different side chains against five different microbes, the OctCl homopolymers were more active against all the microbes than PrCl and HexCl homopolymers, while the OctCl homopolymers showed the highest hemolytic activity. To decrease the polymer toxicity, the polymer composition was varied by copolymerizing different monomers containing varied hydrophobic side chain lengths. By maintaining a charge on each repeat unit but varying the hydrophobicity, polycarbonates with high antimicrobial activity and selectivity were achieved.

## 7. Comparison of Antimicrobial Activity between Polymeric QAS and Polymeric QPS

Both nitrogen and phosphorus atoms are classified in the Nitrogen group in the Periodic Table of the Elements although the polymeric QPS usually show a different performance in emerging applications compared to polymeric QAS due to the intrinsic differences between the nitrogen atom and phosphorus atom. The atomic radius of phosphorus is larger than that of nitrogen, resulting in the lower electronegativity of phosphorus [174]. Therefore, the QPS are weakly-associated cations compared to the corresponding ammonium compounds, which may facilitate the adsorption of QPS onto negatively charged bacterial membranes [175,176,177,178,179].

Various antimicrobial polymeric QPS were prepared by Kenawy *et al.* [180,181] via polymerization of QPS-containing monomers, chemical modification of polymeric precursors, and/or transquaternization on polymers, and showed much higher antibacterial and antifungal activities than the polymeric QAS analogs. A mixture composed of two homopolymers, *i.e.*, poly(tributyl(4-vinylbenzyl)ammonium chloride and poly(tributyl(4-vinylbenzyl)phosphonium chloride, at a ratio of 1:1 exhibited a synergistic effect on antimicrobial performance, which was more effectively biocidal than either the QAS or QPS homopolymer alone. Endo *et al.* proposed that the greater antimicrobial property resulted from the higher solubility of the cellular membrane constituents in the polymeric micelles [182].

El-Newehy *et al.* [183] modified chitosan via immobilization of three types of quaternary onium salts, respectively. The antimicrobial activities of the modified chitosan were evaluated via a cut plug method against bacteria and fungi. With the same counter anion, the QPS-modified chitosan presented higher activities against the tested microbes than the QAS-modified chitosan.

Water-insoluble antimicrobial composite particles containing various QAS or QPS were prepared by surface-grafting styrene onto silica gel particles and subsequently covalently bonding QAS or QPS onto the composite particles [184]. The effect of the structure of functional groups on the antimicrobial efficiency of the modified composite particles was investigated by employing a dynamic shaking flask test against *E. coli*, measuring the content of extracellular DNA/RNA and the activity of TTC (5-triphenyl-2H-tetrazolium chloride) dehydrogenase. The results of all the measurements indicated that the QPS modified composite particles possessed a higher antimicrobial rate and efficiency compared to the QAS modified particles.

A series of biodegradable polyesters with antimicrobial activities were prepared by incorporation of QAS or QPS into the polymeric network [185]. By comparing the MIC values of the cationic compounds and diameters of the inhibition zone induced by the modified polyester disks, the QPS was the second most effective one among the compounds, and the corresponding polyester showed the highest activity compared to the other modified polyesters.

Qiu *et al.* [186] immobilized triphenyl and tributyl QPS onto chlorinated natural rubber, respectively. To compare the antimicrobial activities of QPS and QAS modified rubbers, tributyl QAS modified natural rubber was also prepared. A shaking flask test of the modified rubbers against *E. coli* and *S. aureus* demonstrated that both the QPS-immobilized rubbers were more effectively biocidal than the QAS-immobilized rubber.

## 8. Conclusions and Future Perspectives

Over the course of the past decade, many researchers have made efforts on the development of novel antimicrobial polymeric materials and exploration of their biocidal activities and action modes. In addition to broad-spectrum antimicrobial activity, membrane-active killing mode and easily tuned structures of low-molecular-weight QAS/QPS, polymers containing covalently bonded QAS/QPS possess high and long-term biocidal efficacy with no-leaching of active moieties, and their applications can be expanded by designing and employing different polymeric structures. Nevertheless, there is still a high demand for developing antimicrobial materials aimed at multi-species pathogenic microbes including bacteria, fungi, protozoa, prions and viruses, *etc.* In particular, the non-enveloped viruses, which have no lipid bilayer envelope surrounding the capsid, are very stable and virulent, and difficult to control and/or destroy by conventional antimicrobial materials. Moreover, due to the difficult purification and propensity for oxidation of their precursor phosphines, polymeric QPS have been limited with regard to wide exploration and application; whereas their much higher antimicrobial activity compared to their polymeric QAS analogs suggests that polymeric QPS will have greater prospects for development as antimicrobial agents. Along with the promotion of antimicrobial properties of polymeric QAS/QPS, low cytotoxicity needs to be achieved for the applications where biocompatibility is a decisive factor. Many scientists are currently engaged in investigating structure-bioactivity relationship to achieve an optimal balance between antimicrobial activity and cytotoxicity. Further intensive and systematic studies of such polymeric QAS/QPS are of great interest.
